# Outcomes of Thoracic Surgery in Pediatric Patients: A Single-Center Experience From Al Ribat University Hospital, Sudan

**DOI:** 10.7759/cureus.100227

**Published:** 2025-12-27

**Authors:** Isam Ahmed Abdeljaleel Taha, Mohamed Y Ibrahim, Basma Suliman, Hayat Abuobaida Banagh, Sagad Mohamed, Mohamed Helali, Sami Mohamed Elamin Taha, Musab Mohammed, Nuseiba Abdo, Ishag Nadi Joseph, Rayan Abdalltif Mohamed Seedahmed

**Affiliations:** 1 Pediatric Surgery, Pediatric Surgery Center, National Ribat University Hospital, Khartoum, SDN; 2 Pediatrics and Child Health, Faculty of Medicine, University of Khartoum, Khartoum, SDN

**Keywords:** congenital anomalies, pediatric, sudan, surgical outcomes, thoracic surgery

## Abstract

Background: Pediatric thoracic surgery is a diverse field with a wide variety of congenital and acquired diseases, most of which require complex perioperative care. Knowledge of local trends and outcomes is essential for optimizing surgical care in resource-constrained settings. This project aimed to present the clinical characteristics and surgical outcomes of children who underwent thoracic surgery at Al Ribat University Hospital over five years. This study represents the first comprehensive review of pediatric thoracic surgery outcomes in Sudan, providing valuable baseline data on disease patterns, perioperative complications, and survival in a resource-limited setting.

Methods: This retrospective descriptive study was conducted at the Department of Pediatric Surgery, Al Ribat University Hospital, Sudan. Medical records of all pediatric patients aged 1 day to 16 years who underwent thoracic surgery between March 2018 and March 2023 were examined. Descriptive analysis included frequencies and percentages for demographic data, surgical indications, operative details, postoperative complications, and outcomes.

Results: A total of 418 pediatric patients underwent thoracic surgery, of whom 303 (72.5%) were males. Neonates accounted for 191 patients (45.7%). Congenital lesions were the most common indication for surgery, identified in 286 patients (68.4%), including esophageal atresia/tracheoesophageal fistula (EA/TEF) in 176 patients (61.5%), congenital lobar emphysema in 51 patients (17.8%), and congenital cystic adenomatoid malformation (CCAM) in 18 patients (6.3%). Most procedures were performed through open thoracotomy (392, 93.8%), and intraoperative complications occurred in 51 patients (12.2%). Early postoperative complications developed in 133 patients (32.8%), most frequently respiratory distress in 43 patients (32.3%) and wound infection in 26 patients (19.5%). Overall postoperative mortality was 133 deaths (31.8%), with the highest case fatality rates observed in EA/TEF (89, 50.6%) and thoracic tumors (23, 41.8%).

Conclusion: Congenital anomalies, particularly EA/TEF and congenital lobar emphysema, are the dominant indications for pediatric thoracic surgery in our center. Both EA/TEF and congenital lobar emphysema and tumor conditions carry the highest mortality. Mortality among neonates with complex congenital defects remains high. The findings emphasize the need for enhanced perioperative support, improved neonatal care, and strengthened pediatric surgical services.

## Introduction

The pediatric population presents unique challenges in thoracic surgery due to anatomical peculiarities, physiological immaturity, and a high prevalence of congenital defects [1]. Pediatric thoracic pathology is heterogeneous, with the surgical disease burden varying across different geographic regions [2]. Major congenital thoracic defects, including esophageal atresia and tracheoesophageal fistula (EA/TEF), congenital lobar emphysema, and congenital cystic adenomatoid malformation (CCAM), constitute the primary indications for thoracotomy in children [3-5].

One of the most common and complicated congenital defects encountered in neonates is esophageal atresia and tracheoesophageal fistula [5,6]. The surgical care of EA/TEF has improved significantly, and the survival rate has improved due to the development of the neonatal intensive care unit, perfection of surgical practice, and the knowledge of related malformations. However, the results are still diverse, especially when it comes to centers dealing with high-risk conditions with severe comorbidities [6,7]. Death rates are still high, particularly among patients with concomitant congenital anomalies such as congenital heart disease, sepsis, prematurity, and low birth weight [8,9].

Children with thoracic conditions are normally required to be intervened early to prevent life-threatening complications and achieve the best long-term results. Postoperative complications, including infection, hemorrhage, respiratory distress, post-pneumonectomy syndrome, air leak, pulmonary edema, chylothorax, and recurrent chest infections, are not rare [10]. Prompt management of these complications is prudent, thereby stressing the significance of knowing the risk factors to improve the overall outcomes and guide family counselling. Therefore, outlining the trend and predictors of such complications is inseparable from maximizing the management of perioperative care and enabling the informed counseling of families.

Pediatric thoracic surgery in resource-constrained settings is often challenged by delayed presentation, complex disease profiles, and variability in perioperative care. Despite the clinical importance of this specialty, data describing surgical case mix, management approaches, and outcomes from low-resource countries remain limited. Our single-center experiences may provide useful information about the complexity of cases, clinical management patterns, and variability of outcomes.

The current research aimed to report a five-year experience of outcomes of pediatric thoracic surgery in Al Ribat University Hospital, Sudan. The outcome assessment in this context will provide critical information regarding pediatric thoracic surgery in a resource-constrained environment and supplement the worldwide pool of pediatric surgical outcome information.

## Materials and methods

This was a retrospective descriptive cross-sectional study conducted at the Department of Pediatric Surgery, Al Ribat University Hospital, Sudan. The aim was to evaluate the clinical patterns, operative details, and outcomes of pediatric thoracic surgeries performed over a five-year period (March 2018 to March 2023).

Study population and sample size

The study included all pediatric patients aged 0-16 years who underwent thoracic surgery at Al Ribat University Hospital during the study period. A total of 418 patients met the inclusion criteria and were analyzed. Inclusion criteria comprised all pediatric patients aged 0-16 years, including neonates, infants, children, and adolescents, who underwent thoracic surgical intervention during the five-year study period. Out of the initial records reviewed, 418 patients met these criteria and were included in the analysis. Conversely, the study excluded patients who underwent non-thoracic procedures and those with incomplete or missing medical records that prevented a full assessment of clinical outcomes.

Data collection

Data were extracted from operative theater logbooks, inpatient medical files, and hospital electronic databases. Variables included patient demographics, indications for surgery, associated congenital anomalies, preoperative investigations, intraoperative findings, complications, and early postoperative outcomes.

Data analysis

Data were compiled using Microsoft Excel (Microsoft Corp., Redmond, WA) and analyzed descriptively. Categorical variables were expressed as frequencies and percentages, and continuous variables were presented as means or medians as appropriate.

Outcome measures

The primary outcomes were intraoperative complications, early postoperative morbidity, and mortality within four weeks of surgery. Secondary outcomes included the duration of chest tube drainage and the length of hospital stay.

Ethical considerations

Approval was obtained from the Research and Ethics Committee of Al Ribat University Hospital (approval number: ARUH-ETH/25-11). All patient data were anonymized and handled confidentially in accordance with institutional research policies.

## Results

Baseline characteristics of patient demographics

A total of 418 pediatric patients underwent thoracic surgery during the five-year study period. Of these, 303 patients (72.5%) were males, and 115 patients (27.5%) were females. Neonates (≤30 days) constituted the largest age group with 191 patients (45.7%), followed by infants aged 1-6 months with 68 patients (16.3%) and infants aged 6-12 months with 59 patients (14.1%). Most patients were residents of Khartoum (215, 51.4%), while the remainder (203, 48.6%) came from other regions of Sudan (Table 1).

**Table 1:** Sociodemographic data of the participants

Variable	Category	Frequency	Percentage
Sex	Male	303	72.50%
	Female	115	27.50%
Age groups	1 day to 30 days	191	45.70%
	1 month to 6 months	68	16.30%
	6 months to 12 months	59	14.10%
	1 year to 6 years	25	6.00%
	6 years to 11 years	49	11.70%
	11 years to 16 years	26	6.20%
Residence	Khartoum	215	51.40%
	Center of Sudan	105	25.10%
	North of Sudan	51	12.20%
	Others (west/east/south) of Sudan	47	11.20%

Clinical and preoperative profile

The most frequent presenting symptom was shortness of breath, reported in 348 patients (83.3%), followed by cough/choking in 193 patients (46.2%) and recurrent chest infections in 98 patients (23.4%). Associated congenital anomalies were identified in 160 patients (38.3%), including cardiac anomalies in 93 patients (58.1%), gastrointestinal anomalies in 28 patients (17.5%), and genitourinary anomalies in 19 patients (11.9%). Preoperative intensive care unit (ICU) admission was required in 193 patients (46.2%) (Table 2).

**Table 2:** Clinical profile data of the participants CXR: chest X-ray, CT: computed tomography, MRI: magnetic resonance imaging, ICU: intensive care unit, GIT: gastrointestinal tract, CNS: central nervous system, MS: musculoskeletal, GU: genitourinary

Variable	Category	Frequency	Percentage
Presentation of the patient (total = 569)	Shortness of breath	348	61.20%
	Cough/choking	193	33.90%
	Recurrent chest infection	98	17.20%
	Hemoptysis	5	0.90%
	Foreign body aspiration	3	0.50%
	Corrosive material swallowing	12	2.10%
Congenital associated anomalies (total = 160)	Cardiac	93	58.10%
	GIT	28	17.50%
	CNS	18	11.30%
	MS	2	1.30%
	GU	19	11.90%
Past surgical history	Yes	57	13.60%
	No	361	86.40%
Type of past surgical history	Thoracic surgery	18	31.60%
	Other surgery	39	68.40%
Family history of thoracic surgery	Yes	13	3.10%
	No	405	96.90%
Preoperative images	CXR	418	100.00%
	CT	201	48.10%
	MRI	33	7.90%
	Echo	192	45.90%
	Bronchoscopy	7	1.70%
	Contrast study	45	10.80%
	Esophagoscopy	16	3.80%
Preoperative ICU admission	Yes	193	45.90%
	No	225	53.60%

Indications for thoracotomy

The indications were predominantly congenital, accounting for 286 cases (68.4%), while acquired lesions accounted for 132 cases (31.6%). Among congenital indications, EA/TEF was the most common, occurring in 176 cases (61.5%), followed by congenital lobar emphysema in 51 cases (17.8%), CCAM/congenital pulmonary airway malformation (CPAM) in 18 cases (6.3%), and bronchogenic cysts in 15 cases (5.2%). Less frequent congenital conditions included diaphragmatic eventration in 15 cases (5.2%) and pulmonary sequestration in five cases (1.7%).

Among acquired causes, tumors were the most common indication for thoracotomy, identified in 55 cases (41.7% of acquired lesions), followed by post-pneumonia complications in 48 cases (36.4%). Other acquired conditions included corrosive or perforated esophageal stricture in 14 cases (10.6%), post-chest trauma in eight cases (6.1%), lung hydatid cyst in five cases (3.8%), and airway foreign body in two cases (1.5%) (Table 3).

**Table 3:** Indications and diagnostic distribution of thoracic surgeries EA/TEF: esophageal atresia/tracheoesophageal fistula, CCAM: congenital cystic adenomatoid malformation, CPAM: congenital pulmonary airway malformation

Category	Diagnosis	Frequency	Percentage
Congenital conditions (total = 286)	EA/TEF	176	61.5%
	Lobar emphysema	51	17.8%
	CCAM (CPAM)	18	6.3%
	Bronchogenic cyst	15	5.2%
	Diaphragmatic eversion	15	5.2%
	Sequestration	5	1.7%
	Congenital esophageal stricture	2	0.7%
	Non-function symptomatic lung	2	0.7%
	Esophageal duplication	1	0.3%
	Pericardiac cyst	1	0.3%
Acquired conditions (total =132)	Tumors	55	41.7%
	Post-pneumonia complication	48	36.4%
	Esophageal stricture, corrosive/perforation	14	10.6%
	Post-chest trauma	8	6.1%
	Lung hydatid cyst	5	3.8%
	Foreign body airway	2	1.5%

Details of surgical conditions

In patients with TEF, the lower pouch fistula was the most common site, occurring in 129 patients (92.8% of TEF cases). Associated anomalies were observed in 99 patients (56.3%), most frequently cardiac anomalies in 79 patients (75.2%), followed by renal anomalies in 11 patients (10.5%) and gastrointestinal anomalies in eight patients (7.6%). Most neonates weighed between 2.1 and 3.0 kg at the time of surgery, representing 119 of 176 patients (67.6%) (Table 4).

**Table 4:** Details of EA/TEF (176 patients) EA/TEF: esophageal atresia and tracheoesophageal fistula, GIT: gastrointestinal tract, ARM: anorectal malformation

Variable	Category	Frequency	Percentage
Type	Atresia with TEF	139	79.00%
	Isolated atresia	36	20.50%
	Isolated TEF	1	0.60%
Site of TEF (total = 139)	Upper pouch	3	2.20%
	Lower pouch	129	92.80%
	Both (double TEF)	7	5.00%
Isolated atresia (total = 36)	Single atresia	33	91.70%
	Multiple esophageal atresia	3	8.30%
Association with other anomalies	Yes	99	56.30%
	No	77	43.80%
Associated anomalies (total = 105)	Cardiac	79	75.20%
	Renal	11	10.50%
	ARM+GIT	8	7.60%
	Vertebral	6	5.70%
	Absent radius	1	1.00%
Weight of the patients with EA/TEF	<1 kg	2	1.10%
	1-1.5 kg	7	4.00%
	1.6-2 kg	42	23.90%
	2.1-2.5 kg	29	16.50%
	2.6-3 kg	90	51.10%
	>3 kg	6	3.40%

In patients with congenital lobar emphysema, the left lung was affected in 39 of 51 patients (76.5%), most commonly involving the upper lobe in 27 of those 39 patients (69.2%). Regarding CCAM, 18 cases (100%) were identified, with right-sided lesions in 11 patients (61.1%) and left-sided lesions in seven patients (38.9%). Bronchogenic cysts (15 cases) were more common on the right side in 10 patients (66.7%), compared with the left side in five patients (33.3%). Eventration of the diaphragm was seen in 15 patients (100%).

Post-pneumonia complications leading to thoracotomy were recorded in 48 patients (100%), including lung abscess in 30 patients (62.5%), adhesions in six patients (12.5%), pneumatocele in six patients (12.5%), bronchiectasis in three patients (6.3%), ruptured bullae in two patients (4.2%), and bronchopleural fistula in one patient (2.1%) (Table 5).

**Table 5:** Details of other thoracic conditions CCAM: congenital cystic adenomatoid malformation

Variable	Category	Frequency	Percentage
Congenital lobar emphysema (total = 51)	Right lung	10	19.60%
	Left lung	39	76.50%
	Bilateral	2	3.90%
Upper lobe affected in the left lung cases (total = 39)	Yes	27	69.20%
	No	12	30.80%
Bronchogenic cyst (total = 15)	Left side	5	33.30%
	Right side	10	66.70%
	Bilateral	0	0.00%
CCAM (total = 18)	Right side	11	61.10%
	Left side	7	38.90%
	Bilateral	0	0.00%
Eventration (total = 15)	Right side	10	66.70%
	Left side	5	33.30%
	Bilateral	0	0.00%
Post-pneumonia complications (total = 48)	Lung abscess	30	62.50%
	Adhesions	6	12.50%
	Pneumatocele	6	12.50%
	Bronchiectasis	3	6.30%
	Ruptured bullae	2	4.20%
	Bronchopleural fistula (pneumothorax)	1	2.10%

Tumors were the most common acquired indication for thoracotomy, affecting 55 of 132 acquired cases (41.7%), followed by post-pneumonia complications in 48 patients (36.4%). Other acquired conditions included esophageal stricture secondary to corrosive ingestion or perforation in 14 patients (10.6%), post-chest trauma in eight patients (6.1%), lung hydatid cyst in five patients (3.8%), and airway foreign body in two patients (1.5%).

The types of tumors encountered included neuroblastoma in 25 patients (45.5%), lymphoma in 15 patients (27.3%), teratoma in six patients (10.9%), secondary sarcoma in eight patients (14.5%), and thoracic Wilms tumor in one patient (1.8%). Total resection was achieved in 26 of 55 tumor cases (47.3%), with the remainder 29 cases (52.7%) undergoing biopsy (Table 6).

**Table 6:** Details of thoracic tumors (55 patients)

Variable	Category	Frequency	Percentage
Type of tumor	Neuroblastoma	25	45.50%
	Lymphoma	15	27.30%
	Teratoma	6	10.90%
	Thoracic Wilms tumor	1	1.80%
	Secondary of sarcoma	8	14.50%
Surgery performed	Total resection	26	47.30%
	Biopsies	29	52.70%
Total resection (total = 26)	Neuroblastoma	11	42.30%
	Secondaries	8	30.80%
	Teratoma	6	23.10%
	Wilms tumor	1	3.80%

Operative profile

The majority of procedures were performed via open thoracotomy in 392 patients (93.8%), while thoracoscopy was completed in eight patients (1.9%), and 18 patients (4.3%) required conversion from thoracoscopy to open surgery. The posterolateral incision was the most common approach, used in 309 patients (73.9%), whereas a lateral incision was used in 101 patients (24.2%).

Intraoperative complications occurred in 51 patients (12.2%), including bleeding in 17 patients (33.3%), anastomotic complications in 18 patients (35.3%), and injury to nearby structures in nine patients (17.6%). A total of 12 intraoperative deaths (23.5%) were recorded. The operative duration ranged between one and three hours in 295 patients (70.6%). Blood transfusion was required for 129 patients (30.9%) (Table 7).

**Table 7:** Operative details and intraoperative outcomes

Variable	Category	Frequency	Percentage
Type of surgery	Open thoracotomy	392	93.80%
	Thoracoscopy	8	1.90%
	Thoracoscopy started but converted to open	18	4.30%
Site of incision in thoracotomy	Lateral	101	24.20%
	Posterolateral	309	73.90%
	Anterior	0	0.00%
	Sternotomy	0	0.00%
	Thoracoscopy	8	1.90%
Intraoperative complications	Yes	51	12.20%
	No	367	87.80%
Complications	Bleeding	17	24.60%
	Air leak	6	8.70%
	Failure of esophageal anastomosis in type C	7	10.10%
	Injury to near structure	9	13.00%
	Anastomotic complication	18	26.10%
	Death	12	17.40%
Duration of operation	>1 hour	88	21.10%
	1-2 hours	167	39.90%
	2-3 hours	128	30.60%
	<3 hours	35	8.40%
Blood transfusion	Yes	129	30.90%
	No	289	69.10%

Postoperative course

A chest tube was inserted postoperatively in 406 patients (97.1%), with a duration of <5 days in 255 patients (62.8%) and ≥5 days in 151 patients (37.2%). Postoperative analgesia was provided to all patients, with paracetamol used in 406 patients (100%), non-steroidal anti-inflammatory drugs (NSAIDs) in 200 patients (49.0%), opioids in 48 patients (11.5%), and nerve blocks in 120 patients (29.6%).

Most patients required ICU admission, totaling 271 patients (66.8%), while the remaining 135 patients (33.2%) were managed in the general ward. Mechanical ventilation was required in 93 patients (22.9%).

Early postoperative (<2 weeks) complications occurred in 133 patients (32.8%), including respiratory distress in 43 patients (32.3%), wound infection in 26 patients (19.5%), anastomotic leak in 18 patients (13.5%), pneumothorax in seven patients (5.3%), and postoperative bleeding in three patients (2.3%).

Overall postoperative case fatality within four weeks was 72 patients (17.2%) (Table 8).

**Table 8:** Postoperative management and complications NSAIDs: non-steroidal anti-inflammatory drugs, ICU: intensive care unit

Variable	Category	Frequency	Percentage
Postoperative chest tube insertion	Yes	406	97.10%
	No	12	2.90%
Chest tube duration	<5 days	255	62.80%
	>5 days	151	37.20%
Postoperative pain management	Paracetamol	406	97.10%
	NSAIDs	200	47.80%
	Opioid	48	11.50%
	Nerve block	120	28.70%
	Epidural catheters	0	0.00%
Postoperative admission	ICU	271	66.70%
	General ward	135	33.30%
Postoperative mechanical ventilation	Yes	93	22.90%
	No	313	77.10%
Early postoperative complication (within two weeks)	Yes	133	32.80%
	No	273	67.20%
Complications	Respiratory distress	43	32.30%
	Bleeding	3	2.30%
	Wound infection	26	19.50%
	Pneumothorax	7	5.30%
	Anastomotic esophageal leak	18	13.50%
	Death	36	27.10%

Case fatality

The highest case fatality was observed in esophageal atresia and TEF, with 89 of 176 patients (50.6%) dying postoperatively. This was followed by thoracic tumors, with 23 of 55 patients (41.8%); congenital lobar emphysema, with 10 of 51 patients (19.6%); and diaphragmatic eventration, with three of 15 patients (20%). Other conditions demonstrated lower case fatality rates, as detailed in Table 9.

**Table 9:** Disease-specific deaths EA/TEF: esophageal atresia/tracheoesophageal fistula, CCAM: congenital cystic adenomatoid malformation, CDH: congenital diaphragmatic hernia, FB: foreign body

Disease	Total number of patients	Frequency	Percentage
EA/TEF	176	89	50.60%
Tumors	55	23	41.80%
Lobar emphysema	51	10	19.60%
Post-pneumonia complications	48	2	4.20%
CCAM	18	0	0.00%
Bronchogenic cyst	15	1	6.70%
Eventration CDH	15	3	20.00%
Acquired esophageal stricture for replacement	14	1	7.10%
Post-trauma thoracotomy	8	1	12.50%
Sequestration	5	1	20.00%
Hydatid cyst	5	0	0.00%
Non-function symptomatic lung for pneumectomy	2	1	50.00%
Airway FB removal	2	0	0.00%
Congenital esophageal stricture	2	0	0.00%
Esophageal duplication	1	1	100.00%
Pericardial cyst	1	0	0.00%
Total	418	133	31.80%

## Discussion

This five-year review provides a detailed overview of the indications and outcomes of the pediatric thoracic surgeries performed at Al Ribat University Hospital, Sudan, highlighting the disease spectrum, operative profiles, and outcomes within a resource-limited setting. The predominance of congenital pathologies in this study (286 patients, 68.4%) is consistent with international and regional reports that emphasize congenital anomalies as the major indication for pediatric thoracic operations [3,4,10]. The male predominance (72.5%) and neonatal peak (45.7%) observed in this series align with findings from Ogunleye et al. [3] in Nigeria and Gebreselassie et al. [4] in Ethiopia, both of which identified similar demographic patterns among pediatric thoracotomy patients in low-income countries.

Despite advances in neonatal surgery, the postoperative mortality in this study (89 deaths among 176 patients with EA/TEF, 50.6%) remains significantly higher than the rates reported in high-income centers, where survival exceeds 85%-90% due to improved neonatal intensive care and surgical expertise [8,11]. The high mortality in our cohort may be attributed to delayed presentation, low birth weight, limited access to neonatal ventilatory support, and the presence of multiple congenital anomalies. Cardiac defects were the most common associated anomalies (79 of 105 cases, 75.2%), a finding that echoes the reports of Prasad [9] and Bruch et al., who identified cardiac malformations as key prognostic determinants in EA/TEF [6].

Among the neonates with EA/TOF, the presence of congenital cardiac malformations was a major determinant of outcome. The coexistence of cardiac defects such as a ventricular septal defect, a patent ductus arteriosus, or tetralogy of Fallot significantly compromises postoperative recovery due to hypoxia, hemodynamic instability, and increased risk of anastomotic leak. Keefe et al. [8] and Alansari et al. [7] emphasized that cardiac malformations are the strongest independent predictors of mortality in EA/TEF, regardless of the surgical approach. In Sudan and other low-income settings, this challenge is compounded by the scarcity of neonatal intensive care units, limited access to pediatric anesthesia, and the absence of dedicated pediatric cardiac services. Despite these constraints, the outcomes in this study highlight the remarkable efforts of local surgical teams who managed to achieve acceptable survival rates in a context of severe resource limitations. Strengthening perioperative cardiopulmonary monitoring, expanding pediatric ICU capacity, and developing specialized anesthesia and cardiac surgery training are crucial to further improving survival in this high-risk group.

The predominance of open thoracotomy (392 cases, 93.8%) in this study reflects current practice patterns in many developing countries where thoracoscopic repair remains limited by cost and technical expertise [1,12-16]. Alansari et al. [7] demonstrated in a recent meta-analysis that thoracoscopic repair of EA/TEF offers comparable survival to open thoracotomy with reduced musculoskeletal morbidity and shorter recovery time. Similarly, Gohda et al. [12] and Zhang et al. [16] showed that thoracoscopic repair can be safely performed in neonates of low body weight when performed in specialized centers with appropriate perioperative support. The low utilization of thoracoscopy in this series (eight cases, 1.9%) underscores the urgent need for investment in pediatric minimally invasive surgery in Sudan.

Liu et al. reported excellent survival in infants undergoing lobectomy for congenital lung lesions, especially when performed electively before the onset of severe respiratory distress [11]. In the present series, no deaths (0%) occurred among CCAM cases, highlighting the importance of early surgical intervention and postoperative respiratory management.

The mortality rate among tumor cases (23 of 55 cases, 41.8%) was higher than that reported in developed centers, likely due to advanced diseases at presentation, limited diagnostic imaging, and restricted access to chemotherapy and radiotherapy. Post-pneumonia complications remain a major indication for pediatric thoracotomy in resource-limited settings, a finding comparable to data from Gebreselassie et al. [4].

Hamed et al. observed that airway complications and anastomotic leaks are common after EA/TEF repair and require early bronchoscopic assessment [15]. Di Mitri et al. showed that early use of video-assisted thoracoscopic surgery (VATS) for pleural empyema in children leads to shorter hospital stay, less postoperative pain, and faster recovery [17].

In high-resource settings, mortality rates for pediatric thoracic surgery have fallen below 5% through better neonatal intensive care, early diagnosis, and adoption of minimally invasive surgery [11,12].

The findings of this study are in line with Alemam et al. [18], who reviewed recent global innovations in pediatric thoracic surgery and emphasized the potential of minimally invasive and image-guided approaches to enhance safety and outcomes [19]. Bawazir similarly highlighted the expanding role of thoracoscopy in pediatric patients and its superiority in reducing hospital stay and postoperative morbidity [1]. Transitioning toward such modern techniques in Sudan will require structured training programs, investment in equipment, and regional cooperation with centers experienced in pediatric thoracoscopic surgery.

Despite being limited by its retrospective design and single-center scope, this study provides one of the largest datasets on pediatric thoracic surgery outcomes in Sudan. It underscores the need for strengthening neonatal intensive care units, early detection and referral of congenital thoracic anomalies, and multidisciplinary perioperative management. Establishing specialized training in pediatric thoracoscopy and neonatal surgery could significantly reduce mortality and align outcomes with international standards.

## Conclusions

Congenital anomalies, particularly EA/TEF, remain the leading indication of pediatric thoracic surgery at our center and carry the highest mortality. Improved neonatal care, early referral, and adoption of minimally invasive surgical techniques could markedly improve survival rates. This experience from Al Ribat University Hospital contributes valuable local data to the global understanding of pediatric thoracic surgery outcomes in resource-limited settings and highlights the need for collaborative efforts to advance pediatric surgical care in Sudan and the wider region.
